# Protective effect of leptin against ischemia-reperfusion injury in the rat small intestine

**DOI:** 10.1186/1471-230X-5-37

**Published:** 2005-11-21

**Authors:** Alper Hacioglu, Cem Algin, Ozgul Pasaoglu, Ercument Pasaoglu, Gungor Kanbak

**Affiliations:** 1 Department of General Surgery, Dumlupinar University Hospital, 43270 Kutahya, Turkey; 2 Department of Pathology, Osmangazi University Faculty of Medicine, 26480 Eskisehir, Turkey; 3 Department of General Surgery, Osmangazi University Faculty of Medicine, 26480 Eskisehir, Turkey; 4 Department of Biochemistry, Osmangazi University Faculty of Medicine, 26480 Eskisehir, Turkey

## Abstract

**Background:**

The small intestine is extremely sensitive to ischemia-reperfusion (I/R) injury and a range of microcirculatory disturbances which contribute to tissue damage. Previous studies have shown that leptin plays an important physiological role in the microvasculature. The aim of this study was to evaluate the protective effects of leptin in I/R – induced mucosal injury in the small intestine.

**Methods:**

Forty rats were divided into 5 groups (n = 8). Group I was subjected to a sham operation. Following mesenteric ischemia in group II (control); physiologic saline 1 cm^3^, in group III; leptin 100 μg/kg, and physiologic saline 1 cm^3^, in group IV; *N*^G^-L-arginine methyl ester (L-NAME) 20 mg/kg, and physiologic saline 1 cm^3^, in group V; leptin 100 μg/kg, L-NAME 20 mg/kg, and physiologic saline 1 cm^3 ^were given intra-peritoneally. In these groups, an I/R procedure was performed by occlusion of the superior mesenteric artery for 45 min followed by 120 min reperfusion. After reperfusion, the small intestines were resected for malondialdehyde (MDA) and nitric oxide (NO) concentration and histopathologic properties. Mucosal lesions were scored between 0 and 5. Tissue MDA and NO concentration and histopathologic grades were compared statistically.

**Results:**

Tissue MDA level significantly increased (*P *< 0.05), tissue NO level significantly decreased in group V animals, compared to group III animals respectively (*P *< 0.001). Histopathologically, intestinal injury significantly decreased in the leptin treated ischemic group.

**Conclusion:**

Leptin can be used safely in mesenteric occlusive diseases, since it induces NO formation and release in mesenteric vessels.

## Background

I/R injury is a phenomenon often confronted in surgical pathologies and it is quite important because it causes severe clinical pathologies by causing destruction in close and far tissues. A mean effect of the I/R injury is formed at the reperfusion phase, and free oxygen radicals that appear in the reoxygenized tissue are held responsible for this mechanism [1,2]. NO, recently purported to be endothelium-derivated relaxing factor, reacts with superoxide in pathogical states and might form cytotoxic species including peroxynitrite anion and hydroxyl radical [3]. Generation of the hydroxyl radical could be responsible for some of the injury associated with I/R and other oxidant-mediated disease states [4]. In animal models of I/R injury, administration of nitric oxide synthase (NOS) inhibitors (L-NAME) has been shown to exacerbate intestinal impairment [5]. Furthermore, NO supplementation ameliorates structural and functional damage accompanying experimental I/R [6]. Presently three NOS isoforms, namely NOS-I (neuronal), NOS-II (inducible) and NOS-III (endothelial), have been identified as being responsible for NO formation [7]. NOS-III is the predominant isoform in the gastrointestinal tract [8].

Leptin, a circulating hormone secreted by adipocytes, influences body weight homeostasis through effects on food intake and energy expenditure [9,10]. Invitro studies showed that leptin has the potency to mediate mitogenic effects on endothelial cells [11-13], monocytes [14], respiratory epithelial cells [15], glomerular endothelial cells [16], adrenal cells [17] and colonic epithelial cells [18]. Moreover, leptin accelerates the healing of colonic anastomoses [19]. Recently, it has been reported that the functional leptin receptor OB-Rb is expressed in endothelial cells and it is functionally competent [20]. This provides evidence that the endothelium is a target for leptin action [21]. Previous studies showed that leptin controls the release of NO by activating NOS [22].

We hypothesize that leptin stimulates NO production and release from the small intestine mesenteric endothelium. L-NAME and leptin were used, and tissue MDA, NO levels were measured.

The aim of this study was to assess the possibility of leptin treatment in the mesenteric occlusive diseases and to investigate the relationship between leptin and NO in intestinal I/R injury in Wistar rats.

## Methods

All experiments were performed in the Surgical Research Center at Osmangazi University. The University Ethics Committee approved this study. Adult male Wistar rats weighting 200 and 250 g housed in a cage were given standard rat feed with free access to water. Rats were anesthetized with thiopentone sodium (Pental, Bayer) and heparinized with sodium heparin (Nevparin, Mustafa Nevzat) intraperitoneally after a 12-h starvation period. After the abdominal wall was cleansed with 10% povidone-iodine (Betadine, Seton), laparatomy was performed by a midline incision. All rats were divided into five groups.

Group I (n = 8); ***sham operated group***

Group II (n = 8); ***I/R group***: following 45 min mesenteric ischemia, given 1 cm^3 ^physiologic saline intraperitoneally

Group III (n = 8); ***I/R+Leptin group***: following 45 min mesenteric ischemia, given 100 μg/kg leptin, and 1 cm^3 ^physiologic saline intraperitoneally

Group IV (n = 8); ***I/R+L-NAME group***: following 45 min mesenteric ischemia, given 20 mg/kg L-NAME, and 1 cm^3 ^physiologic saline intraperitoneally

Group V (n = 8); ***I/R+Leptin+L-NAME group***: following 45 min mesenteric ischemia, given 100 μg/kg leptin, 20 mg/kg L-NAME, and 1 cm^3 ^physiologic saline intraperitoneally

**Mesenteric ischemia **was performed in groups II-V. Small intestine was subjected to 45 min of ischemia achieved by occluding of superior mesenteric artery with a vascular clamp, and at the end of the ischemia, 120 min reperfusion was performed.

100 μg/kg of **Leptin **(Sigma-Aldrich, Germany) was given intraperitoneally as defined previously [23] at the end of the mesenteric ischemia in groups III, V. The aim of leptin administration at the end of ischemia, was to assess the treatment properties of this hormone on reperfusion injury.

**L-NAME **(Sigma-St.Louis, USA) was given 20 mg/kg intraperitoneally as defined previously [23] at the end of the mesenteric ischemia in groups IV, V.

After occlusion of superior mesenteric artery with a vascular clamp, the laparotomy incision was closed continuously with 3/0 silk (Silk, Ethicon, UK) sutures in all groups. The rats were placed in their cages. 45 min after their operation, relaparotomy was performed following the same anaesthesia protocol and vascular clamp was removed. 120 min reperfusion was performed and reperfusion of the mesenteric vasculature was confirmed by the return of pulsation of the vascular arcade.

The entire small intestine was carefully removed and placed on ice. The small intestine was divided into two equal segments: the proximal (jejunum) and distal (ileum). Each segment was rinsed thoroughly with physiologic saline and opened longitudinally to expose the intestinal epithelium. The jejunum segment was separated into two equal pieces, and one of them was used to determine the tissue MDA and NO concentration, and was immediately placed in deep freezer at -70°C.

### Determination of MDA

Freezed intestinal tissue was homogenized 1:10 in 1.15% KCI, and MDA levels were determined according to tiobarbituric acid method [24]. Results were expressed as nmol MDA/mg protein.

### Determination of NO

Nitrate in tissue was assayed by a modification of the cadmium-reduction method as Cortas et al. defined [25]. The nitrite produced was determined by diazotization of sulphanylamide and coupling to naphthylethylene diamine. After samples were deproteinized with Somogyi reagent, the nitrate was reduced by Cu-coated Cd in glycine buffer at pH 9.7 (2.5 to 3 g of Cd granules for a 4 ml reaction mixture). The reduction followed pseudo-first-order reaction kinetics, a convenient time interval for assay being 90 min. Maximum reduction occurred at about 2 h. Results were expressed as pmol/mg protein.

### Histopathologic examination

Ileum segments were placed in 10% formalin solution. Paraffin blocks were prepared from the tissue pieces and were kept in 10% formalin solution. The sections with 4.5 micron thickness were painted with Haematoxylin-Eosin (HE). Light microscopic studies were reviewed by a pathologist blinded to protocol. The degree of intestinal tissue injury was evaluated and each was graded from 0 to 5, identical to that originally described by Chiu et al [26]. Grade 0 was defined as normal mucosa and grade 1 was the development of a subepithelial space at the tips of the villi. This space was more extended in grade 2. And in grade 3 there was a massive epithelial lifting down the sides of the villi. In grade 4, the villi were denuded of epithelium and in grade 5; it was characterized by a loss of the villi themselves.

### Statistical analysis

Data was presented as mean ± standard derivation (SD). One-way analysis of variance (ANOVA) and Tukey Post Hoc (parametric) test was used to compare tissue MDA and NO levels between the groups. Histopathologic grades of the groups were compared with Kruskal-Wallis test and Tukey Post Hoc (nonparametric) test. The differences were considered significant at *P *< 0.05.

## Results

### Evaluation of mesenteric ischemia

**Figure 1:**
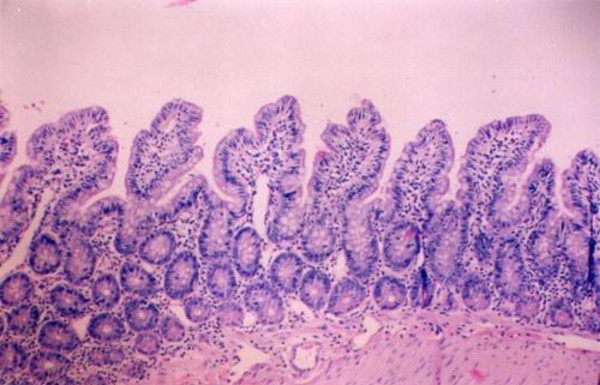
Normal intestinal mucosa in group I rats (Grade 0) (HE, ×100).

**Figure 2:**
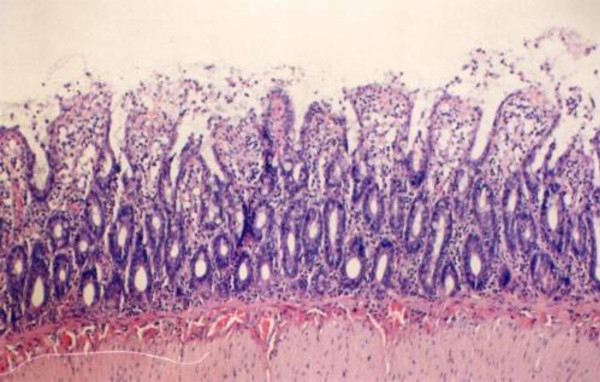
Intestinal villus after I/R in group II rats. Note lifted epithelium, white blood cells, and monocytes in the Lamina propria (Grade IV) (HE, ×100).

Tissue MDA and NO levels significantly increased in ***I/R group ***animals, compared to ***sham operated group ***animals (MDA: 3.76 ± 0.17 vs. 2.54 ± 0.09 nmol/mg, *P *< 0.05; NO: 201.00 ± 11.59 vs. 126.75 ± 12.38 pmol/mg, *P *< 0.05) (Table 1). On histopathological analysis, intestinal tissue injury significantly increased in ***I/R group***(Figure 2) animals, compared to ***sham operated group***(Figure 1) animals (Jejunum: 3.45 ± 0.17 vs. 0.00 ± 0.08, χ^2 ^= 28,63 df = 4 *P *< 0.001; ileum: 3.73 ± 0.13 vs. 0.00 ± 0.00, χ^2 ^= 28,63 df = 4 *P *< 0.001) (Table 2).

### Effects of leptin on mesenteric ischemia

**Figure 3:**
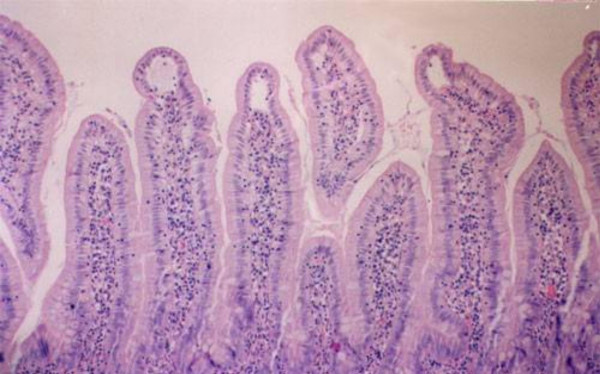
Development of Gruenhagen's space at tip of a villus in group III rats (Grade II) (HE, ×100).

Tissue MDA level significantly decreased in ***I/R+Leptin group ***animals, compared to ***I/R group ***animals (MDA: 2.80 ± 0.18 vs. 3.76 ± 0.17 nmol/mg, *P *< 0.05), but tissue NO level significantly increased in ***I/R+Leptin group ***animals, compared to ***I/R group ***animals (NO: 345.38 ± 22.29 vs. 201.00 ± 11.59 pmol/mg, *P *< 0.001). On histopathological analysis, intestinal tissue injury significantly decreased in jejunum in ***I/R+Leptin group ***(Figure 3) animals, compared to ***I/R group ***animals (2.71 ± 0.08 vs. 3.45 ± 0.17, χ^2 ^= 28,63 df = 4 *P *< 0.05). However, there was no significant difference in intestinal injury in ileum between ***I/R+Leptin group ***and ***I/R group ***animals (Table 2).

### Effect of L-NAME on mesenteric ischemia

**Figure 4:**
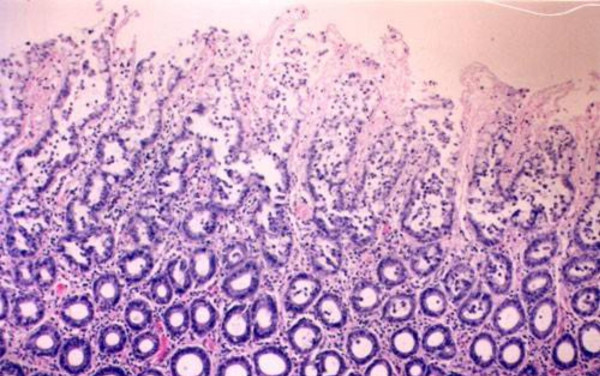
Mucosal ulceration, and necrosis with invasion of muscularis propria in group IV rats (Grade V) (HE, ×100).

Tissue MDA level increased in **I/R+L-NAME group **animals compared to ***I/R group ***animals (MDA: 3.88 ± 0.22 vs. 3.76 ± 0.17 nmol/mg, *P *> 0.05), but tissue NO level significantly decreased in ***I/R+L-NAME group ***animals, compared to ***I/R group ***animals (NO: 140.25 ± 8.81 vs. 201.00 ± 11.59 pmol/mg, *P *< 0.05). On histopathological analysis, there was no significant difference in intestinal injury between ***I/R+L-NAME group ***(Figure 4) and ***I/R group animals ***(Table 2).

### Effects of leptin and L-NAME on mesenteric ischemia

**Figure 5:**
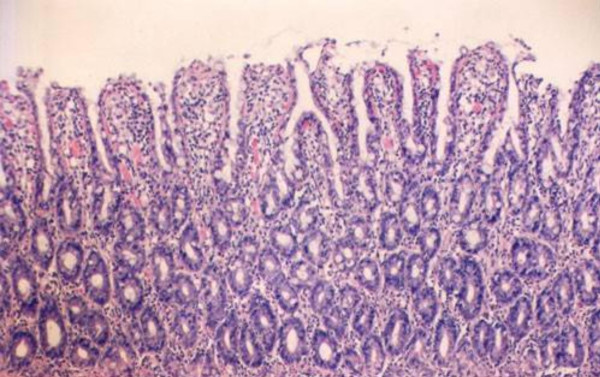
Villus necrosis involving >distal one-half villus involvement in group V rats (Grade III) (HE, ×100).

Tissue MDA level significantly increased in ***I/R+Leptin+L-NAME group ***animals, compared to ***I/R+Leptin group ***animals (MDA: 3.74 ± 0.20 vs. 2.80 ± 0.18 nmol/mg, *P *< 0.05) there was no significant difference in tissue MDA levels between ***I/R+Leptin+L-NAME group ***and ***I/R+L-NAME group***. Tissue NO level significantly decreased in ***I/R+Leptin+L-NAME group ***animals, compared to ***I/R+Leptin group ***animals (NO: 183.50 ± 14.63 vs. 345.38 ± 22.29 pmol/mg, *P *< 0.001), but there was no significant difference in tissue NO level between ***I/R+Leptin+L-NAME group ***and ***I/R+L-NAME group***. On histopathological analysis, intestinal tissue injury significantly increased in ***I/R+Leptin+L-NAME group ***(Figure 5) animals, compared to ***I/R+Leptin group ***animals (Jejunum: 3.44 ± 0.20 vs. 2.71 ± 0.08, χ^2 ^= 28,63 df = 4 *P *< 0.05; ileum: 3.63 ± 0.14 vs. 2.88 ± 0.20, χ^2 ^= 28,63 df = 4 *P *< 0.05). However, there was no significant difference in intestinal injury between **I*/R+Leptin+L-NAME group ***and ***I/R+L-NAME group ***animals (Table 2).

## Discussion

Syndromes of mesenteric ischemia remain clinically challenging, despite decades of surgical experience. The causes of mesenteric ischemia are well known and its major complication, gangrenous necrosis of portions of the gastrointestinal tract, is well recognized by all surgeons. Nevertheless, diagnosis and effective treatment is often delayed in these patients, and when gut infarction occurs, the mortality rates range from 50% to 80%, making mesenteric ischemia one of our most lethal vascular problems [27]. Ischemia and consecutive reperfusion causes oxidative stress, which is characterized by an imbalance between reactive oxygen species (ROS) and the anti-oxidative defence system. Reperfusion of ischemic tissue, although necessary for a reparative mechanism, has been shown to worsen acute ischemic injury via the release of ROS [28]. I/R injury to the small intestine causes local production of the ROS which are known to play an important role in gut epithelial damage [29]. Increased lipid peroxidation in lung, liver and small intestine in animals with intestinal I/R, was evidenced by significantly increased MDA levels in all three organs. This previously has been demonstrated in different studies with significantly increased MDA levels in lung [30] and intestine, induced by intestinal I/R [31]. Recent evidence has suggested that NO may play a significant role in the maintenance of mucosal integrity. In the mesenteric endothelium, low levels of continuous release of NO by NOS III, is thought to be a major determinant of vascular tone and regulator of blood flow of the mucosa [32]. In the present study, 45 min of mesenteric ischemia was achieved by the occlusion of superior mesenteric artery with a vascular clamp and 120 min reperfusion was regained after ischemia. This study also showed that, tissue MDA and NO levels significantly increased in ischemic groups. On a histopathological analysis, intestinal tissue injury significantly increased in the ischemic group, compared to the non-ischemic group.

Leptin, a circulating hormone secreted by adipocytes, influences body weight homeostasis through effects on food intake and energy expenditure [9,10]. Previous studies showed that leptin increases NO synthesis in a dose-dependent manner in Wistar rats [33]. Kimura et al. showed that leptin relaxes the rat mesenteric artery. The vasodilatation of mesentery beds is mediated by endothelium-derivated NO [34]. It is well known that NO is produced by NOS in endothelial cells [35]. It relaxes vessels by stimulating guanylate cyclase in underlying smooth muscle cells [36]. In our study, tissue MDA level significantly decreased in leptin-treated ischemic group, compared to the ischemic group, but tissue NO level significantly increased in the leptin-treated ischemic group, compared to the ischemic group. On histopathological analysis, intestinal tissue injury significantly decreased in the leptin-treated ischemic group, compared to the ischemic group. This results supports the findings of Kimura et al.

In animal models of I/R injury, administration of NOS inhibitors was shown that these exacerbate intestinal impairment [5]. NO synthesis is inhibited by L-arginine analogues, such as L-NAME [37]. In our study, tissue MDA level increased in the L-NAME-administrated ischemic group, compared to the ischemic group, but tissue NO level significantly decreased in the L-NAME-administrated ischemic group, compared to the ischemic group. On histopathological analysis, intestinal tissue injury increased in the L-NAME-administrated ischemic group, compared to the ischemic group.

Salzman et al showed that in the mesenteric endothelium, low level continuous release of NO by NOS III is thought to be a major determinant of vascular tone and regulator of blood flow of the mucosa [32]; and previous studies showed that leptin plays a major role in NO release by activating NOS in the adipocytes and the adjacent capillary endothelium [22]. Kimura et al showed that leptin relaxes the rat mesenteric artery. The relaxation is mediated by nitric oxide released from endothelium, and Cl(-) plays an important role in leptin-induced nitric oxide release [34]. In our study, tissue MDA level significantly increased in the L-NAME-administrated, leptin-treated group, compared to the leptin-treated group, but tissue MDA level decreased in the leptin-treated group, compared to the L-NAME-administrated group. Tissue NO level significantly decreased in the L-NAME-administrated, leptin-treated group, compared to the leptin-treated group, but increased in L-NAME-administrated, leptin-treated group compared to L-NAME-administrated group, but there was no statistical difference. On histopathological analysis, intestinal tissue injury significantly increased in the L-NAME-administrated, leptin-treated ischemic group, compared to the leptin-treated ischemic group, but decreased compared to the L-NAME-administrated ischemic group. This results indicates that leptin was unable to increase the tissue NO levels in animals, subjected to NOS inhibition.

The leptin gene was isolated by Friedman's group, through positional cloning from ob/ob mice that failed to produce leptin and displayed extreme obesity and hyperphagia. There is high degree of homology in leptin from different species [38]. Obesity in both rodents and humans is generally associated with elevated leptin levels [39]. Another form of mouse obesity, designated db/db, demonstrates symptoms similar to those observed in ob/ob mice, but the db/db mice failed to be rescued by leptin administration. It was proposed that db/db mice might have a mutation of the gene encoding the leptin receptor [40]. The genes for both leptin and the leptin receptor have been cloned and sequenced from the mouse, rat and human, as well as from bovine, porcine and ovine tissues [38]. In most human obesity cases [41], as well as in model animals e.g. mice [42], obese individuals displayed elevated plasma leptin levels compared to subjects of normal weight, thus demonstrating "leptin resistance". These findings demonstrate that leptin plays an important role in obesity and metabolic disorders, like diabetes.

Recently Lin et al. has reported that leptin has a time-dependent response and orexin-A has a delayed response to acute inflammatory stimuli such as intestinal I/R injury and they may participate in metabolic disorders in injury as inflammatory cytokines. In rats, subjected to 60 minutes of ischemia they found that, after 30 minutes of reperfusion serum leptin levels decrease significantly and after 360 minutes of reperfusion they increase significantly; compared to the serum leptin levels before injury and to sham-operated animals after injury [43]. On the other hand, previously published studies demonstrate that, at the onset of reperfusion tissue NO levels decrease [44]. This study showed that the stimulatory effect of leptin on NO synthesis and release significantly decreased under NO synthesis inhibition by L-NAME.

## Conclusion

This study envisages the possibility that leptin can be used safely in mesenteric occlusive diseases, since it induces NO production and release in mesenteric vessels.

## List of abbreviations

I/R: Ischemia-reperfusion,

L-NAME: *N*^G^-L-arginine methyl ester

MDA: Malondialdehyde

NO: Nitric oxide

NOS: Nitric oxide synthase

HE: Haematoxylin-Eosin

SD: Standard derivation

ANOVA: One-way analysis of variance

ROS: Reactive oxygen species

## Competing interests

The author(s) declare that they have no competing interests.

## Authors' contributions

A.H. participated in the experimental studies and in the preparation of the manuscript.

C.A. contributed to the design of the study and critically reviewed the manuscript

O.P. participated in the histopathological studies.

E.P. coordinated the study.

G.K. participated in the biochemical studies.

## Pre-publication history

The pre-publication history for this paper can be accessed here:



## Figures and Tables

**Table 1:** Effects of leptin on tissue MDA and NO levels

**Groups **(n = 8)	**Tissue MDA **(nmol/mg protein)	**Groups**				
		1	2	3	4	5

I	2.54 ± 0.09					
II	3.76 ± 0.17	*				
III	2.80 ± 0.18		*			
IV	3.88 ± 0.22	*		*		
V	3.74 ± 0.20	*		*		
	**Tissue NO **(pmol/mg protein)					
I	126.75 ± 12.38					
II	201.00 ± 11.59	*				
III	345.38 ± 22.29	*	*			
IV	140.25 ± 8.81		*	*		
V	183.50 ± 14.63			*		

* *P *< 0.05

Tissue MDA: F_(4:35) _= 12,60 *P *< 0.001; ANOVA. *P *< 0.05; Tukey Post Hoc (parametric) Test

Tissue NO: F_4:35 _= 35,25 *P *< 0.001; ANOVA. *P *< 0.05; Tukey Post Hoc (parametric) Test

**Table 2:** Intestinal injury scores in groups

**Groups **(n = 8)	**Jejunum **(Median score)	**Groups**				
		1	2	3	4	5

I	0.00± 0.08					
II	3.45± 0.17	*				
III	2.71± 0.08	*	*			
IV	3.71± 0.16	*		*		
V	3.44± 0.20	*		*		
	**Ileum **(Median score)					
I	0.00± 0.00					
II	3.73± 0.13	*				
III	2.88± 0.20	*				
IV	3.94± 0.18	*		*		
V	3.63± 0.14	*		*		

* *P *< 0.05

Jejunum: χ^2 ^= 28,63 df = 4, *P *< 0.001; Kruskal-Wallis Test. *P *< 0.05; Tukey Post Hoc (nonparametric) Test

Ileum: χ^2 ^= 25,88 df = 4, *P *< 0.001; Kruskal-Wallis Test. *P *< 0.05; Tukey Post Hoc (nonparametric) Test
